# The relevant range of scales for multi-scale contextual spatial modelling

**DOI:** 10.1038/s41598-019-51395-3

**Published:** 2019-10-15

**Authors:** Thorsten Behrens, Raphael A. Viscarra Rossel, Ruth Kerry, Robert MacMillan, Karsten Schmidt, Juhwan Lee, Thomas Scholten, A-Xing Zhu

**Affiliations:** 1 Eberhard Karls University Tuebingen, Cluster of Excellence “Machine Learning: New Perspectives for Science”, Maria von Linden Str. 6, 72076 Tuebingen, Germany; 2 Eberhard Karls University Tuebingen, Department of Geosciences, Soil science and Geomorphology, Ruemelinstrasse 19-23, 72070 Tuebingen, Germany; 3 Soil & Landscape Science, School of Molecular and Life Sciences, Faculty of Science and Engineering, Curtin University, Perth, WA 6845 GPO Box U1987, Australia; 4 Brigham Young University, Department of Geography, 690 Spencer W. Kimball Tower, Provo, UT 84602 USA; 5 LandMapper Environmental Solutions Inc., 702-250 Douglas Street, Victoria, BC Canada; 6 University of Wisconsin-Madison, Department of Geography, Madison, WI53706 USA; 7 The Chinese Academy of Sciences, State Key Laboratory of Environmental Information Systems, Institumte of Geographical Sciences and Natural Resources, Beijing, 100101 China

**Keywords:** Environmental sciences, Geomorphology

## Abstract

Spatial autocorrelation in the residuals of spatial environmental models can be due to missing covariate information. In many cases, this spatial autocorrelation can be accounted for by using covariates from multiple scales. Here, we propose a data-driven, objective and systematic method for deriving the relevant range of scales, with distinct upper and lower scale limits, for spatial modelling with machine learning and evaluated its effect on modelling accuracy. We also tested an approach that uses the variogram to see whether such an effective scale space can be approximated a priori and at smaller computational cost. Results showed that modelling with an effective scale space can improve spatial modelling with machine learning and that there is a strong correlation between properties of the variogram and the relevant range of scales. Hence, the variogram of a soil property can be used for a priori approximations of the effective scale space for contextual spatial modelling and is therefore an important analytical tool not only in geostatistics, but also for analyzing structural dependencies in contextual spatial modelling.

## Introduction

Environmental properties are the result of complex and non-linear interactions of physical, chemical and biological processes across space and time. Often, these interactions are so complex that the spatial variation of the environmental property cannot be explained deterministically. Geostatistical methods treat variation as if it were random in terms of spatially autocorrelated random fields or random processes^1,2^. This is why associations between the spatial patterns of kriged predictions and the physical processes that influence and control these patterns often remain hidden^3^.

The universal model of spatial variation introduced by Matheron^4^ describes the variation of a soil property *Z*(*s*) in terms of a deterministic component Z^*^(*s*) consisting of structural variation, a stochastic component *ε*′(*s*) consisting of (apparently) random variation that may be spatially correlated and *ε*′ consisting of spatially uncorrelated random noise:

$$Z(s)={Z}^{\ast }(s)+\varepsilon ^{\prime} (s)+\varepsilon ^{\prime} $$

The structural variation of the deterministic component Z^*^(*s*) can be modelled by correlation between the target variable and other environmental variables following the concept of Jenny^5^. With such models, for example, soil properties are expected to be predictable in terms of their correlation to environmental covariates that represent the factors of soil formation: climate, organisms, relief, and parent material, as they influence the dominant soil forming processes at any location. The stochastic component *ε*′(*s*) consisting of spatially correlated variation is often modelled with kriging^6^.

Recent studies have shown that the use of multiple scales of covariates in environmental correlation models can increase prediction accuracy^7–15^. Some of these contextual modelling approaches also facilitate pedological interpretation^10–12,16^. The increase in prediction accuracy, compared to methods that are not multi-scale, is understood to be related to the fact that the spatial variation observed in soil properties occurs simultaneously at many scales^3,9,16,17^ in response to soil-forming processes that themselves vary across multiple scales and are captured and described at multiple spatial resolutions^9,16^. It has been shown that modelling can directly, as well as indirectly, account for relevant spatial contextual influences stemming from interacting, nested, hierarchical and scale dependent processes^9–11,16,18,19^. However, it remains challenging to efficiently unravel the effective scale space for modelling^20^. Moreover, being able to estimate the effective scale space in advance of the modelling would be advantageous to help determine if multi-scale modelling is necessary and to improve the parsimony, accuracy and interpretability of the modelling^16^.

The aims of this paper are to (i) present a method that derives the effective scale space, with defined lower and upper scale space limits, for contextual spatial modelling with machine learning and (ii) derive an a priori approximation of the effective scale space, using the variogram. Our hypothesis is that using covariates that encompass only the effective scale space is economic in terms of computational cost and provides parsimonious and interpretable models with prediction accuracies that are at least as good as a model that uses all scales.

To analyze the influence of environmental covariates that operate over multiple scales, terrain derivatives computed from Gaussian pyramid octaves of a Digital Elevation Model (DEM) were used^11,12^. The analysis is based on four different soil datasets.

## Methods

### Gaussian pyramid mixed scaling

Gaussian mixed scaling^11,12^ was used to prepare the multi-scale terrain derivatives used for modelling. The Gaussian pyramid is a multi-scale signal processing method based on Gaussian filtering and down-sampling^21^. It can be used to decompose the scales of environmental covariates^11^. Each down-sampling step reduces the cell size by half while the Gaussian filter helps to reduce associated artifacts. During each down-sampling step every second row and every second column is removed from the raster dataset. The output of a down-sampling step is called an octave. For environmental modelling these octaves are ultimately up-sampled, i.e. interpolated, back to the original resolution, to ensure that all covariates used possess the same cell size as the original covariate^11^. In mixed scaling, the DEM is down-sampled to all possible octaves. Then, the terrain attributes are calculated for each octave and finally the terrain derivatives are up-scaled back to the resolution of the original DEM. Compared to other scaling approaches, mixed scaling has been demonstrated to produce the best prediction accuracy while still being interpretative^12^.

The following terrain attributes were calculated at each scale of a Gaussian pyramid based on the equations presented by Zevenbergen and Thorne^22^:

- elevation
- slope
- sine transformed aspect
- cosine transformed aspect
- average curvature
- profile curvature
- planform curvature

### Machine learning and validation

The Random Forests approach, as implemented in R^23,24^, was used as the machine learning model. Random Forests have been used in various pedometric studies over the past decade^9,25–29^ as well as in many other environmental assessments^30,31^. The modelling accuracy was evaluated with 10 times, 10-fold cross-validation using the caret package for R^32^.

### Extracting the relevant range of scales based on contextual modelling

We developed additive and subtractive multi-scale models based on mixed scaling to analyze the influence of different scales on the spatial modeling^12^. This approach is exhaustive in that it iterates successively with the covariates from individual scales. For the additive models, coarser scale covariates were successively included in models and the prediction accuracy of each model, described by the coefficient of determination (*R*^2^), was computed. For the subtractive models, finer scale covariates were removed in succession from the entire set of scales, starting from the finest, and the *R*^2^ was computed after each step. Previous studies, including multi-scale analyses, have shown that, with the additive approach, the maximum prediction accuracy is achieved only after multiple coarser scales have been added^8–12^. With the subtractive approach, prediction accuracy is expected to be good at the start, because, at first, all scales are included in the model. But accuracy can be seen to decrease only slightly if finer scale covariates, which can represent noise rather than signal, are removed. Consequently, the maximum effective scale required for modeling can be identified when the accuracy in the additive approach no longer improves. Similarly, the minimum scale can be identified when prediction accuracy in the subtractive approach first begins to show a significant decrease. For a given data set, a combination of both the additive and subtractive analyses should make it possible to identify and select the most relevant range of scales of the covariates.

### Variography

In geostatistics, the variogram is used to develop a theoretical model from empirical data that describes the degree of spatial autocorrelation of an environmental property. The parameters of the variogram model are the nugget, sill and range. The nugget describes the small-scale variability of the data and measurement errors^33,34^ that appear spatially random at the scale of investigation and is the y-intercept of the variogram. The maximum variability between point pairs is represented by the sill. The nugget:sill ratio therefore represents the degree of spatial dependency. Smaller nugget:sill ratios indicate greater proportions of spatially dependent variation. The range of the variogram is the maximum distance up to which a soil property is spatially autocorrelated. Hence, it may be considered an indicator for the maximum scale of the contextual environmental processes relevant to soil formation. Several studies have suggested investigating the relationship between the variogram and structural dependencies of environmental covariates^3,9,20,35,36^.

### Extracting the relevant range of scales based on the variogram

We used the range of the variogram of a soil property of interest to define the upper limit of the relevant range of scales and the nugget:sill ratio multiplied by the range to approximate the lower limit of the relevant range of scales. This lower limit approximation is based on the assumption that the variability of the soil property at the short-scale, as represented by the nugget effect^34^, can be approximately converted into a spatial scale. i.e. if the nugget is relatively large, resulting in weak spatial dependence, then the influence of fine-scale covariates should also be small. Conversely, if the nugget is relatively small, i.e. there is strong spatial dependence, then the fine-scale covariates should improve the prediction accuracy of spatial models. If they were absent, the small-scale differences could not be accounted for in the modelling.

The separation distance up to which point pairs are included in semivariance estimates of the variogram (cutoff) was extended, from the length of the diagonal of the box spanning the data divided by three (the default used by gstat^37^), to 85% of the diagonal length. We did this because the scales analyzed with the machine learning approach are relatively large and partially exceed the extent of the area covered by samples, for the study sites.

To optimally describe the relevant range of scales by properties of the variogram, we used anisotropic variograms based on eight different directions. The smallest and the largest values from all estimates of the lower and upper limit of the effective scale space were used to define the relevant range. The following angles were used to derive the anisotropic variograms: 0°, 22.5°, 45°, 67.5°, 90°, 112.5°, 135° and 157.5°. Variograms that could not be fitted automatically, which might be due to a too small set of samples or singularities in the model, were ignored.

The variogram properties strongly depend on the theoretical variogram model. Here the spherical model was used for all cases in order to increase interpretability. In this respect, the spherical model shows the most meaningful and interpretable values for nugget, sill and range. The variogram models were automatically fitted with the gstat package^37^ in R to avoid any subjective influences on fitting.

### Moran’s I

We used Moran’s I (*MI*)^12,38^ to test the efficacy of contextual modelling for reducing or eliminating spatial auto-correlation in the residuals at all steps of the additive and subtractive modelling. The *MI* ranges from −1 to 1, where full dispersion is indicated by −1, randomness by 0, and clustering by 1, respectively.

### Correlating lag and scale

**Figure 1:**
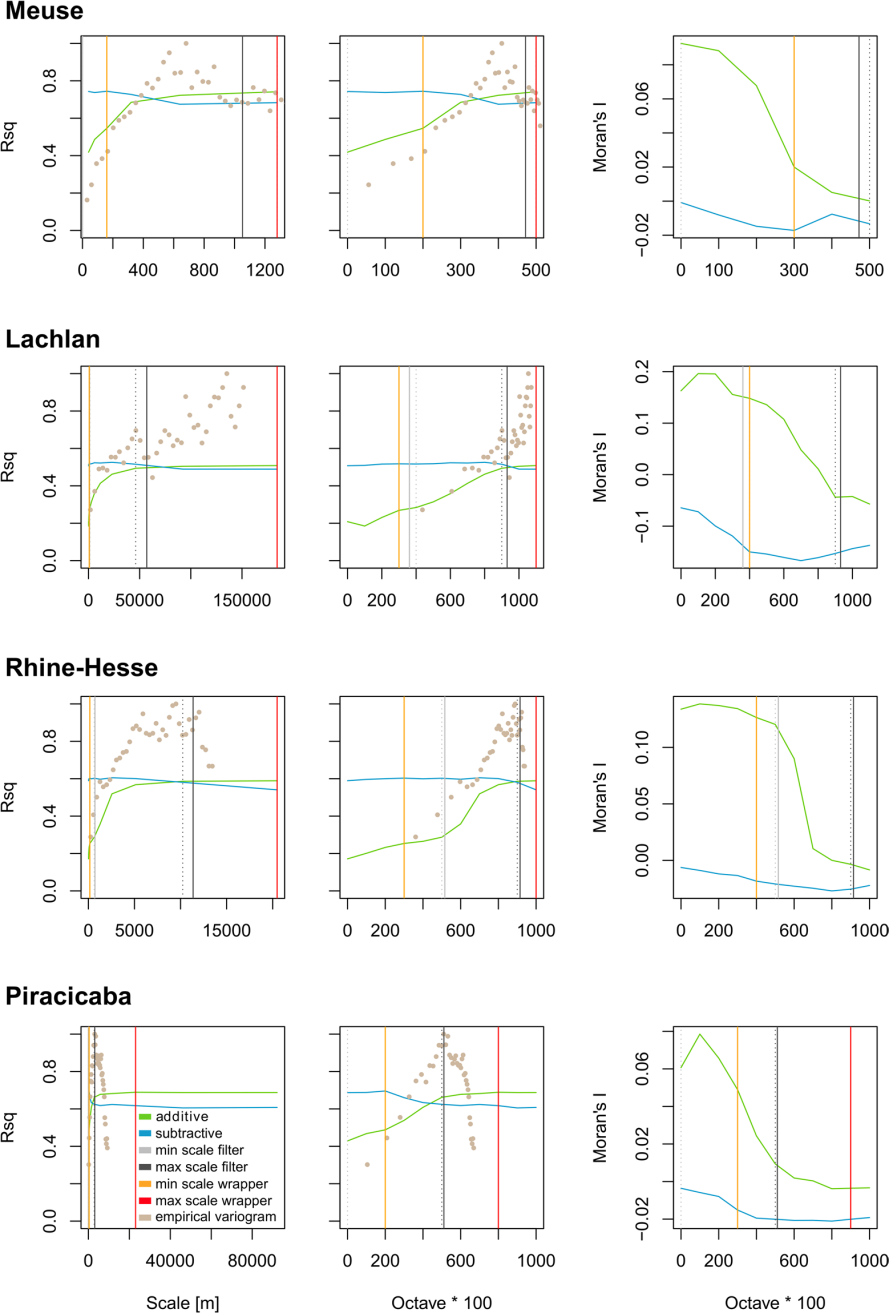
The first and second columns show the contextual machine learning results for the scale in meters and the respective Gaussian pyramid octaves, while the third column shows the corresponding Morans’I values. The green line represents the additive and the blue line the subtractive approach. The relevant scale range determined by the contextual machine learning method is marked by orange and red vertical lines representing the lower and upper limits of the effective scale space. The corresponding variographically determined limits are displayed in light and dark grey. The dashed lines show the octave closest to these values which were used for modeling. The normalized (0–1) experimental variograms are also shown for both the scales and the octaves in the respective transformations.

**Figure 2:**
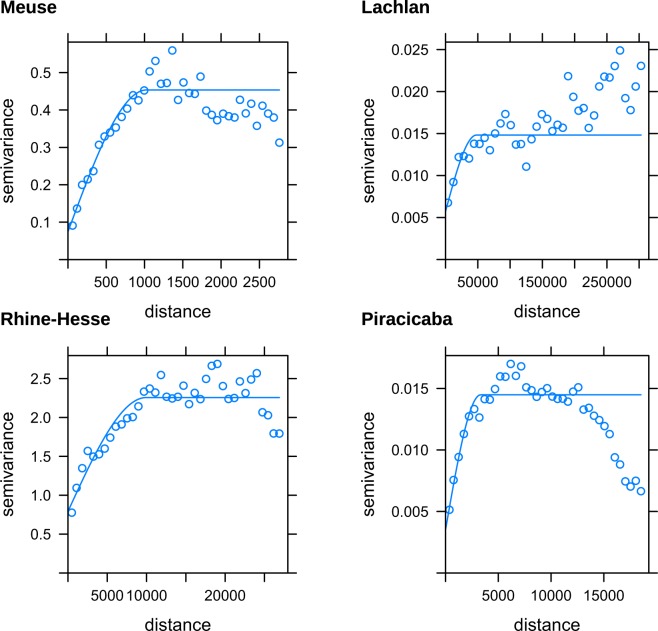
Spherical isotropic variograms of the soil properties for the four study sites. The properties of the isotropic variograms are shown in Table 1.

The scale in the Gaussian scale space is described by the pixel size, which is halved with each step when creating the pyramid. In contrast, the lag of the variogram represents a radius. A lag distance of e.g. 10 m corresponds to a spatial support of 20 m and thus twice the pixel size of a pyramid octave of 10 m. Therefore, the lag distances of the variogram were divided by a factor of 2 to obtain a common basis for the analysis of scales. These converted values are shown in Fig. 1 for the empirical variogram, while the variograms in Fig. 2 are based on the original lag distance.

### Datasets

#### Meuse

Heavy metal distribution across the Meuse floodplain is driven by polluted sediments carried by the river and preferentially deposited close to the river bank and in areas with lower elevation. The Meuse dataset consists of 155 samples from the River Meuse floodplain (The Netherlands)^39,40^. In this study log-transformed zinc concentration and a 40 m DEM were used.

#### Lachlan

The Lachlan dataset comes from an agricultural production and grazing area on the Lachan River Catchment in central western New South Wales, Australia. The climate ranges from sub-alpine to semi-arid conditions with rainfall ranging from 280 mm to over 1000 mm, while the geology in the catchment area is complex and has a significant impact on the soil. Modelling was based on using 300 samples of bulk density and terrain covariates computed from an SRTM DEM with 90 m resolution.

#### Rhine-Hesse

The Rhine-Hesse (Germany) data set is an example of a dataset with a strongly autocorrelated distribution of soil properties^9,12^. Samples (n = 342) of the top-soil silt content (0–10 cm) were used. The spatial distribution of silt content is influenced by wind erosion and loess translocation from the Rhine-Main lowlands to the surrounding heights of Rhine-Hesse. The silt content was transformed using sqrt(max(silt) - silt) and a 20 m DEM was used.

#### Piracicaba

The Piracicaba study area describes a sugarcane growing region in Brazil^10,12^. Soil samples (n = 321) of topsoil clay content (0–10 cm) were used for modelling. Soil formation patterns strongly reflect those of the underlying rock formations, strike and dip and subsequent erosion due to a relatively high precipitation. The clay content was transformed using sqrt(clay). An SRTM DEM with a resolution of 90 m was used.

## Results

### Contextual multiscale modeling

For all four study sites, successive addition of coarser scales of DEM-based covariates (additive) to a random forest model generally increased prediction accuracy, while successive removal of finer scales (subtractive) from the model generally decreased prediction accuracy after a certain point (Fig. 1, first two columns). The increase, as well as the decrease, in prediction accuracy is not linear and shows some discontinuities. In the additive approach, the Lachlan data set shows a decrease in the explained variance (*R*^2^) when the predictors representing the second scale are added to the predictors of the original (finest) scale. For the Piracicaba data set, adding the largest scale covariates results in a decrease in prediction accuracy. With the subtractive approach, the *R*^2^ generally starts to decrease at a certain scale, when relevant information represented by fine to medium scale covariates is excluded from the model. In all examples, however, it was noted that the *R*^2^ increases when the finest scales, containing irrelevant and noisy information, were removed.

**Figure 3:**
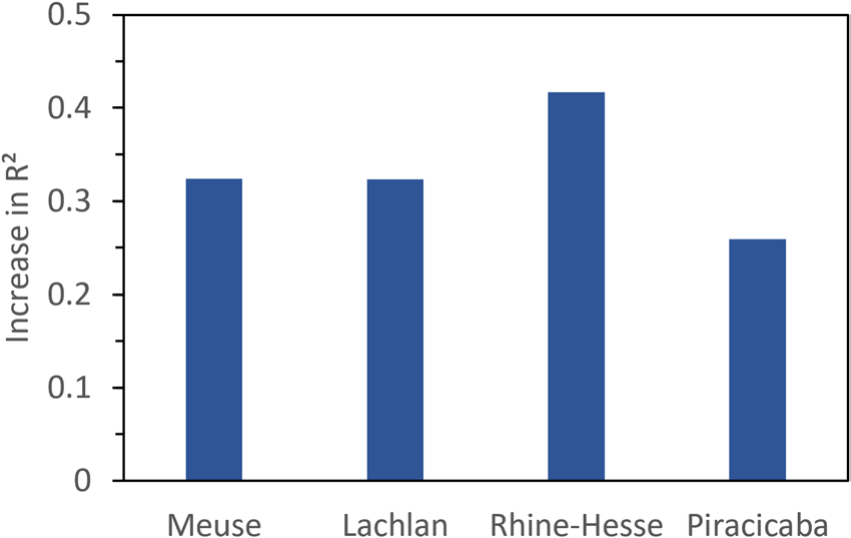
Increase in cross-validated prediction accuracy using all scales compared to the covariates only at the original (finest) scale.

Compared to modeling using only the original (finest) scale covariates, the average increase in the explained variance when all scales were included is 33% (Figs 1 and 3). The smallest increase is for the Piracicaba data (26%) and the largest for the Rhein-Hesse data (42%). The decrease of *R*^2^ for the subtractive model is considerably smaller for all study areas, with an average decrease of 5.2% (Fig. 1).

### Moran’s I

The diagrams of Moran’s I are based on the residuals of the additive and subtractive models. They show that the continuous addition of coarser scales ultimately leads to complete disappearance of autocorrelation in the residuals (Fig. 1, right column). However, when the very coarsest scales were included, negative autocorrelation was sometimes observed to occur. This effect is pronounced for the subtractive approach, where fine scales are removed from the dataset.

Similar to the analysis of the modelling accuracy, some discontinuities are visible, especially at the finer scales, i.e. with the addition of a coarser scale, the spatial autocorrelation can increase as revealed for the Lachlan and Piracicaba data sets.

### Variography

Figure 2 shows variograms of the soil properties for the different study sites and Fig. 1 shows the empirical variograms overlaid on the results of the additive and subtractive models. The figure highlights the good correspondence between the variogram and the effective scale space derived with the additive and subtractive approach.

**Figure 4:**
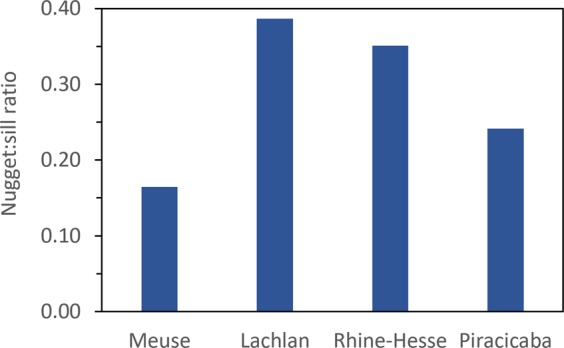
Nugget:sill ratio of the isotropic variograms for the four study sites.

The variogram parameters are shown in Table 1, while the nugget:sill ratio is shown in Fig. 4. The nugget:sill ratio is largest for the Lachlan and Rhine-Hesse data sets. The anisotropic variograms are shown in the third column of Fig. 2 and are based on the default cutoff value, for better interpretation of the finer scales.

**Table 1:** Properties of the isotropic spherical variograms of the soil properties of the different datasets.

Dataset	Nugget	Sill	Range	Nugget/sill * range
Meuse	0.075	0.454	1026	169
Lachlan	0.006	0.015	47926	18516
Rhine-Hesse	0.792	2.257	9966	3497
Piracicaba	0.003	0.014	3334	805

Table 2 compares the minimum and maximum scales derived using the isotropic and anisotropic variograms. The range of the anisotropic variogram can be more than double the range of the isotropic variogram (Piracicaba). The minimum scale of the isotropic variogram is up to 23 times larger than the minimum scale of the anisotropic variogram. The smallest value of the minimum scale and the largest value of the range of the anisotropic variograms were used to define the relevant range of scales.

**Table 2:** Lower and upper boundaries of the effective scale space based on isotropic and anisotropic variograms of the soil properties.

Dataset	Minimum scale		Maximum scale	
	isotropic	anisotropic	isotropic	anisotropic
Meuse	84	30	513	677
Lachlan	9258	395	23963	34041
Rhine-Hesse	1748	299	4983	7660
Piracicaba	402	246	1667	4175

The minimum scale is calculated by multiplying the nugget:sill ratio with the range of the variogram and the maximum scale equals the range of the variogram. For the anisotripic variograms the overall minimum and maximum scales were selected. The orgininal variogram distances were divided by a factor of 2 to obtain a common basis for the analysis of scales with the Gaussian scale space (see Methods).

### The relevant range of scales

For most study sites, the minimum scale estimated using variography is similar to the minimum relevant range established using the subtractive modelling approach (on average, about one scale different). The maximum anisotropic scale, estimated from the variograms, corresponds well with the point at which the curve of the additive approach begins to flatten out, which is close to the maximum scale derived from the additive approach. This can also be seen when comparing the experimental variogram to the additive and subtractive approach (Fig. 1). Although, on average, the maximum range derived with variography is about 2 scales smaller than the results of the additive and subtractive approach, there is relatively good and consistent agreement between the effective scale space determined with both approaches.

**Figure 5:**
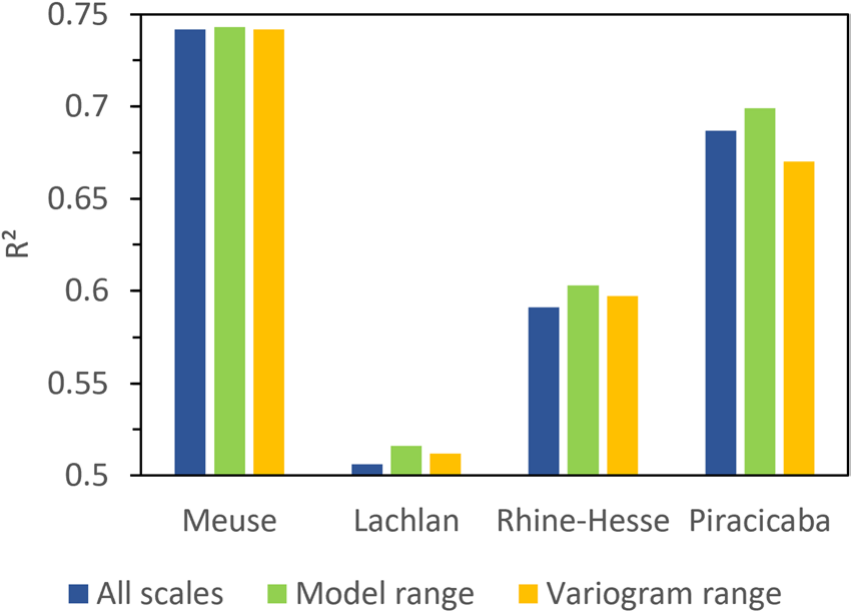
Comparison of the influence of selecting the relevant range based on the contextual machine learning method (green) and the variography method (yellow) with the results for the full range of scales (blue).

In all cases, the first two or three scales (octaves) of DEM derived covariates seem to contain noisy or irrelevant predictors. For the Piracicaba data set, the coarsest investigated scale is also irrelevant (Fig. 1). Figure 5 compares the prediction accuracies across the entire range of scales, the relevant range of scales derived using the machine learning models, as well as the relevant range estimate based on variography. Although the increase is relatively small, in all cases, prediction accuracy was best when only covariates representing the effective scale space were used. Except for the Piracicaba site, using the variogram to estimate the effective scale space a priori leads to an increase in the explained variance compared to using data from all scales.

## Discussion

It is important to identify the appropriate scale space in spatial modelling to improve parsimony, computational efficiency, and to remove noise and augment interpretability. We presented two approaches by which the relevant range of scales for spatial modelling may be identified. One is exhaustive and derives the relevant range using data-driven machine learning with different sets of multi-scale DEM-based covariates added and removed incrementally. The second approach is based on an analysis of the properties of the variogram derived from point data of the soil property being modelled. The first is accurate but computationally demanding, while the latter allows for the relevant range of scales to be approximated a priori and is relatively rapid to derive. Using the variogram, one could determine if multi-scale modelling is necessary. Depending on the size of the dataset this can therefore reduce modelling time with machine learning by hours or even days.

Although there is a good correspondence between the two approaches to identify the relevant range of scales, the range of scales as determined by the variogram does not lead to an increase in the explained variance in all cases. This effect can be traced back to the generally longer-range scales identified by the Gaussian pyramid compared to the lower cutoff value determined by variogram. Additionally, non-linear interactions of covariates at very coarse scales may lead to non-stationary effects that cannot be explained by variography. If, however, one considers the empirical variograms, i.e. the raw data from the sample set and not the fitted variogram function, the optimum range could well be greater, in many cases, compared to the automatically fitted values. The latter would lead to larger, and more appropriate, maximum ranges for some datasets. For this reason, we recommend that the empirical variogram should also be considered when trying to determine the relevant range from the variogram for the soil property.

The non-linearities observed towards the endpoints of the additive and subtractive models (Fig. 1) may be due to either noise, irrelevant information or effects of multicollinearity, which reduces the accuracy of the models. With successive removal of finer scales, the *R*^2^ generally drops, while the MI increases above the value of the entire data set. This shows that the fine scales contain noise, which is the reason why selecting a reduced range of scales can lead to higher prediction accuracies. Interestingly, when including the very coarsest scales, negative autocorrelation can occur. Generally, little is known about negative spatial autocorrelation and especially the consequences of negative spatial autocorrelation for regression-based inference^41^. What can be seen from the subtractive model, is that the negative spatial autocorrelation effect gets stronger when fine scales are removed. This could be an effect of missing fine to medium scale information, which, when approximated from non-linear combinations of the coarser scale covariates in the machine learning models, leads to the negative spatial autocorrelation.

The considerably smaller decrease of *R*^2^ in the subtractive models, compared to larger increases in the additive models, confirms that coarser scales are often more informative than those of finer scale covariates. This effect has also been observed in previous studies^10,11^, including the multi scale analyses of soil spectra^42^. The minimum scales required to achieve the most accurate model results are often coarser than the original (finest) scale of the selected (DEM) environmental predictors. This also supports similar findings from previous studies^8,10,15,42^. This is related to the effective scales of the physical processes which influence the development of soil properties, but may also be related to sampling density and sampling error. Higher density samples, taken at shorter distances apart, and covariates with finer resolutions may extend the relevant range of scales and thus help to better estimate the pedologically relevant range of scales.

Finally, the significance of our work is that we have shown that there is a good relationship between spatial dependence, as described by the variogram of a soil property, and the relevant scale space (maximum and minimum effective resolutions) in contextual spatial modelling with machine learning. Thus, the variogram of a soil property can be used as an analytical tool to inform multiscale contextual spatial modelling using machine learning. This method should have a broader appeal as it can also be used to describe the appropriate scales for modelling other environmental phenomena, for example in land-management^20^, or in ecology to, describe animal habitat relationships^43^.

## Data Availability

The Meuse data set that supports the findings of this study is available through the R package sp^40^. The other datasets were used under license for the current study, and thus are not publicly available. Data are however available from the corresponding author upon request depending on the permission of the licensors.
